# Virtual Transitions and Opportunities in LTSS education Post-Pandemic

**DOI:** 10.1093/geroni/igab046.406

**Published:** 2021-12-17

**Authors:** Gail Towsley, Jacqueline Telonidis, Cherie Brunker, Linda Edelman

**Affiliations:** 1 University of Utah College of Nursing, Salt Lake City, Utah, United States; 2 University of Utah College of Nursing, Salt lake City, Utah, United States; 3 University of Utah School of medicine, Salt Lake City, Utah, United States

## Abstract

The Utah Geriatric Education Consortium Learning Community transitioned to the Age-Friendly Long-Term Services and Support (LTSS) ECHO with support from Comagine Health, our local QIN-QIO. ECHO sessions utilize case-based learning and mentorship to help community providers gain the expertise required to provide needed care and/or services to older adults. Since March 2020, and in response to the needs of our partners, four ECHO sessions (average of 47 attendees per session) have focused on COVID-19 training including COVID-19 briefings, infection prevention, positive thinking and coping with stress. With our partners, we also co-created a 3-part LTSS telehealth ECHO series to illustrate how telehealth can address the unique challenges of COVID-19. We will discuss 1) how we met the educational needs of our partners during a health crisis 2) the process we took to develop the LTSS telehealth ECHO series, and 3) opportunities for continued virtual education application.

